# Agro-Industrial By-Products of Plant Origin: Therapeutic Uses as well as Antimicrobial and Antioxidant Activity

**DOI:** 10.3390/biom14070762

**Published:** 2024-06-26

**Authors:** Yessica Enciso-Martínez, B. Shain Zuñiga-Martínez, Jesús Fernando Ayala-Zavala, J. Abraham Domínguez-Avila, Gustavo A. González-Aguilar, Manuel Viuda-Martos

**Affiliations:** 1 Coordinación de Tecnología de Alimentos de Origen Vegetal, Centro de Investigación en Alimentación y Desarrollo, A.C., Carretera Gustavo Enrique Astiazarán Rosas, La Victoria 46, Hermosillo 83304, Sonora, Mexico; yenciso220@estudiantes.ciad.mx (Y.E.-M.); bzuniga221@estudiantes.ciad.mx (B.S.Z.-M.); jayala@ciad.mx (J.F.A.-Z.); jesus.dominguez@ciad.mx (J.A.D.-A.); gustavo@ciad.mx (G.A.G.-A.); 2 IPOA Research Group, Agro-Food Technology Department, Instituto de Investigación e Innovación Agroalimentaria y Agroambiental (CIAGRO-UMH), Universidad Miguel Hernández, 03312 Alicante, Spain

**Keywords:** agro-industrial wastes, antioxidants, bioactive compounds, functional properties

## Abstract

The importance of bioactive compounds in agro-industrial by-products of plant origin lies in their direct impacts on human health. These compounds have been shown to possess antioxidant, anti-inflammatory, and antimicrobial properties, contributing to disease prevention and strengthening the immune system. In particular, the antimicrobial action of these compounds emerges as an important tool in food preservation, providing natural alternatives to synthetic preservatives and contributing to combating antimicrobial resistance. Using agro-industrial by-products of plant origin not only addresses the need to reduce waste and promote sustainability but also inaugurates a new era in the formulation of functional foods. From fruit peels to pulps and seeds, these by-products are emerging as essential ingredients in the creation of products that can promote health. Continued research in this area will unveil new applications and properties of these by-products and open doors to a food paradigm in which health and sustainability converge, paving the way to a healthier and more equitable future. The present review presents an overview of our knowledge of agro-industrial by-products and some of their more relevant health-promoting bioactivities.

## 1. Introduction

Worldwide, the agro-food industry produces over 190 million tons of by-products yearly [1]. Fruits, vegetables, roots, and tubers represent 40% to 50% of these by-products, which are commonly relegated to landfills or inappropriate sites [2]. Agro-industrial by-products of plant origin are generated at all points of the supply chain, including agricultural production, processing, and distribution. They mainly comprise unusable pulps, peels, seeds, pods, stems, and shells derived from food processing [3]. Due to their composition, they degrade slowly, leading to their accumulation and a negative environmental impact [4]. However, transforming them into value-added products could reduce losses by increasing their demand and become an essential strategy for developing sustainable industrial processes [5]. Moreover, agro-industrial by-products of plant origin contain considerable concentrations of bioactive compounds (such as phenolic compounds and carotenoids), which possess multiple functionalities and bioactivities, making them useful as potential ingredients in various food products [6,7].

These bioactive compounds can provide health benefits beyond their nutritional value, and since they remain in food matrices, most are found in agro-industrial by-products [8]. They can therefore be used to prevent and treat diseases like dyslipidemia, diabetes, and cancer as well as exert neuroprotection. Additionally, when incorporated into foods, these compounds can enhance their nutritional, sensory, and technological properties [9], making them a promising, economically attractive, and sustainable alternative.

These bioactive compounds can also act effectively against pathogenic bacteria. Studies on the antimicrobial properties of these by-products and their bioactivities have focused on the effect of different extracts against Gram-positive and Gram-negative bacteria and characterizing the profile of the active compounds [10]. Using extracts from agro-industrial by-products with antimicrobial activity has emerged as a critical strategy for creating natural alternatives to synthetic chemicals, opening new possibilities for identifying and applying them in different sectors, ranging from medicine to agriculture.

The novelty of this manuscript lies in the integration of updated scientific evidence that examines how these by-products are not only rich in antioxidants, like phenolics and flavonoids, which help neutralize free radicals and reduce cellular oxidative stress, but also how these and other compounds that exert antimicrobial activity can inhibit the growth of pathogenic microorganisms. In addition, it explores how incorporating these by-products into one’s diet can significantly contribute to the prevention of chronic diseases, thus strengthening the scientific basis for their use in functional food products and innovative pharmaceutical formulations. These topics are often discussed separately; however, the bioactive profile of by-products allows them to simultaneously counter pathogens, exert antioxidant effects, and improve consumer health.

The present review aims to provide a comprehensive overview of the potential of agro-industrial by-products of plant origin, highlighting their therapeutic uses and their antioxidant and antimicrobial activities. The main bioactive compounds present in these by-products are identified, their effectiveness in the prevention and treatment of various diseases is discussed, and their applicability in various industries is discussed.

## 2. Bioactive Compounds in Agro-Industrial By-Products

Bioactive compounds have significant potential applications, and agro-industrial by-products constitute a valuable and economically feasible source of these compounds [8]. Research on their chemical and functional characterization has revealed diverse bioactive compounds that can add benefits to food products, while also benefitting consumers [11]. There is also significant variability in recovered bioactive components, which is closely related to the species, variety, and plant tissue. These aspects have been widely studied to maximize their yield. For example, in the fruit industry, the generation of pomace is significant; this by-product has been shown to contain levels of bioactive compounds comparable to those of fruit juices. Additionally, industrial processing of popular vegetables, such as potatoes, tomatoes, and carrots, produces by-products that can be used in the formulation of food products while also exerting antimicrobial and antioxidant effects due to the presence of hydrolyzed peptides, thereby extending the shelf life of the food [12].

Particularly noteworthy among these bioactive compounds are phenolic compounds, which have attracted great interest due to their multiple applications in the food and pharmaceutical industries. These compounds are distinguished by their varied bioactive properties, including antihypertensive, antimicrobial, and antioxidant activities, which improve consumers’ overall health [13]. The versatility of phenolic compounds has been capitalized on in various industries, including the food, pharmaceutical, and cosmetics industries [14,15]. In various by-products like peels, seeds, flowers, leaves, stems, pomace, and bagasse, the compounds of interest will vary, for example, some may be rich in phenolic acids, stilbenes, and flavonoids, while others may have a significantly different profile and concentrations. The diversity of bioactive compounds in agro-industrial by-products highlights their value and potential valorization [16]; however, utilizing them at larger scales requires a thorough knowledge of their composition and bioactivities.

Research has been conducted to harness these bioactive compounds in the search for new alternatives for utilizing agro-industrial by-products. For example, studies on cocoa residues, such as mucilage and bean shells, have revealed the presence of compounds of considerable interest. In an analysis of cocoa mucilage samples, specifically of the National x Trinitarian type, a remarkable total phenolic content of 105.08 mg gallic acid equivalent (GAE)/100 mL was observed, as well as a total flavonoid content of 36.80 mg catechin equivalent (CE)/100 mL. Specific compounds like catechin (35.44 mg/L), procyanidins (B2: 35.10; B1: 25.68; C1: 16.83 mg/L), and epicatechin (13.71 mg/L) were also reported. Regarding cocoa bean shells (the CCN-51 variety), they have been shown to contain notable levels of total phenolics (42.17 mg GAE/100 g) and total flavonoids (20.57 mg CE/100 g) [17]. These data highlight the potential of cocoa by-products as a significant source of phenolic components, suggesting their potential application as functional ingredients in the food industry. The phenolic acids and flavonoids contained in them can exert bioactivities that have various benefits for human health; for example, it has been demonstrated that a moderate consumption of these bioactive compounds exerts antioxidant and antimutagenic effects. Such bioactivities are due to the prevention or delayed oxidation of other molecules, such as nucleic acids, lipids, carbohydrates, and proteins, which helps reduce the impact of free radicals in the body. This, in turn, lowers the risk of non-communicable diseases like cancer, diabetes, and cardiovascular diseases [18].

Another agro-industrial by-product of great interest is alperujo, which is generated during the extraction of olive oil, and constitutes the semi-solid fraction of this process. Several compounds have been identified and quantified in this by-product, including hydroxytyrosol, hydroxytyrosol 4-β-d-glucoside, 3,4-dihydroxyphenylglycol, and tyrosol [19]. These compounds exhibit various biological activities, including antioxidant, anti-inflammatory, immunomodulatory, anticancer, antithrombotic, antimicrobial, and antifungal. Thus, alperujo has excellent potential to be employed in the food and pharmaceutical industries; additionally, its utilization increases the efficient use of olives and promotes sustainable practices in the olive industry [20].

Pomegranate processing is accompanied by a high generation of by-products. A study by Toledo-Merma et al. [21] focused on the valorization of some of the more representative ones, including its peel and carpellar membranes; the authors report phenolic compounds like α-punicalagin (48.22 mg/100 g dry weight (d.w.)), β-punicalagin (146.58 mg/100 g d.w.), and ellagic acid (25.57 mg/100 g d.w.). These molecules could be extracted and used as natural ingredients with preservative properties, antioxidants, and other functionalities in various food applications.

A study by Abbasi-Parizad et al. [18] focused on grape residues, coffee grounds, tomato residues, and red corn cobs, which were collected to analyze and characterize the phenolic acids, flavonoids, and anthocyanins present in them. The results highlighted a remarkable variability in their total phenolic content, ranging from 4.64 to 22.77 mg GAE/g d.w. Regarding grape pomace, flavonoids (17,186 μg/g d.w.) account for more than 95% of its total phenolics, while also containing quercetin, apigenin, and naringenin. As for phenolic acids in this by-product (785 μg/g d.w.), there is a predominance of gallic (282 μg/g d.w.) and ellagic (247 μg/g d.w.) acids. The composition of coffee bagasse stands out for its concentrations of gallic acid (272 μg/g d.w.) and chlorogenic acids (592 μg/g d.w.). In the case of tomato pomace, the phenolic acids extracted (978 μg/g d.w.) are mainly composed of cinnamic acid, *p*-coumaric acid, and caffeic acid. Flavonoids make up 65% of the total phenolic compounds in tomato pomace, with the presence of naringenin and naringenin chalcone standing out. Finally, red corn cob has a phenolic acid content (3050 μg/g d.w.) that represents 29% of its total phenolics, with chlorogenic acid (1233 μg/g d.w.) and ferulic acid (712 μg/g d.w.) being the most abundant ones. Its main flavonoids include catechin (2887 μg/g d.w.) and epicatechin (1739 μg/g d.w.). Anti-inflammatory assays (IL-8 gene expression in Caco-2 cells) of the aforementioned by-products showed that grape pomace and spent coffee grounds exhibited the best results. This anti-inflammatory activity was attributed to the high flavonoid content in the by-product, with other phenolic compounds playing a minor role.

Cantaloupe melon stands out as one of the most consumed melons worldwide; however, its industrial processing generates considerable amounts of by-products like peels and seeds. To evaluate their potential, a study conducted by Vella et al. [22] focused on analyzing the peels’ total phenolic content, which was reported as 25.48 mg GAE/g, and the seeds’ total phenolic content, which was 1.50 mg GAE/g. Phenolics are present in both edible and inedible parts of plants, including melon by-products, with flavonoids standing out in the peel (15.19 mg CE/g), as compared to the seeds (0.74 mg CE/g). When analyzing the tannin content, a higher content was observed in the peels (11.83 mg GAE/g), as compared to the seeds (0.92 mg GAE/g). These results suggest that cantaloupe melon by-products are a significant source of phenolics, with various potential industrial applications. Considering these properties opens new perspectives for the reuse and valorization of melon by-products, which contributes to their sustainable consumption.

Although phenolic compounds have a variety of biological properties that make them relevant for different industries, their antioxidant capacity is their most prominent one. They can act as natural antioxidants that mitigate oxidative stress in consumers, due to disrupting autooxidation chain reactions and inhibiting the production of free radicals, among other related functions [23]. In line with emerging market trends, studies have shown that some phenolic compounds can be incorporated into various food matrices to strengthen them, thus increasing their nutritional content [24].

Carotenoids are another group of bioactive compounds in agro-industrial by-products that are of significant interest. Their chemical structures allows these compounds to perform vital functions in the human body, which are closely associated with their antioxidant properties, effectively eliminating peroxyl and other radicals [25]. Since the human body cannot synthesize them, carotenoids are obtained from one’s diet, which has led to numerous investigations to take advantage of natural carotenoid sources, including agro-industrial by-products, which represent an economical and sustainable option to obtain them. For example, research by Gea-Botella et al. [26] shows a high carotenoid content from persimmon pulp and skin of 2444.54 mg/100 g extract. Similarly, Lara-Abia et al. [27] extracted carotenoids and carotenoid esters from papaya pulp and peel using soybean oil, obtaining 59 μg carotenoids/g oil. Szabo et al. [28] proposed the recovery of carotenoids present in tomato peels, which yielded 1.5 mg of lutein, 40.5 mg of lycopene, and 4.9 mg of β-carotene, with a total sum of 46.9 mg/100 g (d.w.). Lima et al. [29] obtained extracts with a high carotenoid content from guava by-products by maceration, totaling 79.04 mg/100 g of total carotenoids. Using agro-industrial by-products as carotenoid sources may be an economically viable option to enrich commonly consumed foods; these findings therefore support the feasibility of recovering and reusing carotenoid-rich by-products, a practice that also contributes to a circular economy.

Agro-industrial by-products of plant origin are rich in various bioactive compounds; Table 1 summarizes some of the main compounds present in various by-products, as well as their reported concentration [30,31,32,33,34,35,36,37].

## 3. Antimicrobial Action of Agro-Industrial By-Products of Plant Origin

The bioactive compounds present in agro-industrial by-products of plant origin play a crucial role in their potential applications; their antimicrobial action stands out due to its ability to inhibit the growth of pathogenic microorganisms that can contribute to food spoilage and are detrimental to consumer health. The bioactive profile of by-products derived from industrial processing is diverse; thus, their expected antimicrobial actions are expected to vary. The effectiveness of a given by-product must therefore be determined in order to establish the doses at which it is effective; moreover, the compounds responsible for such bioactivities should also be determined. Phenolics, flavonoids, and hydrolyzed peptides have shown remarkable antimicrobial efficacy [38], making them potential candidates with which this bioactivity can be associated.

The antimicrobial action of said compounds is due to several mechanisms; for example, phenolics and flavonoids can destabilize bacterial cell membranes, altering their permeability and functionality, while also inhibiting bacterial growth or promoting cell lysis. These compounds can also interfere with enzymes essential for microbial replication and metabolism, reducing the viability of pathogens [39]. Hydrolyzed peptides in by-products also contribute to antimicrobial activity by inserting themselves into bacterial membranes, forming pores that allow essential ions and nutrients to leak out, leading to cell death. Some peptides can also penetrate the interior of microbial cells and alter critical metabolic processes [40].

The antimicrobial efficacy of bioactive compounds is closely related to their chemical structure and concentration. Studies have shown that certain phenolics and flavonoids possess increased antimicrobial activity due to hydroxyl groups that facilitate the generation of reactive oxygen species (ROS). These ROS induce oxidative stress in microbial cells, damaging lipids, proteins, and DNA, inhibiting cell growth or death [41]. In the food industry, incorporating agro-industrial by-products rich in bioactive compounds into food formulations can improve product safety and quality. These compounds act as natural preservatives, extending the shelf life of food by inhibiting the growth of pathogens. They can also reduce the need for synthetic additives, promoting a more natural and sustainable approach.

Research by Giordano et al. [42] used the extract of kiwi (*Actinidia deliciosa* cv ‘Hayward’) peels to determine the antimicrobial activity of this by-product against various pathogenic bacteria. The results showed a similar minimum inhibitory (MIC) and bactericidal (MBC) concentration against *Staphylococcus aureus* and *Escherichia coli* (MIC 1 mg/mL, MBC 2 mg/mL). Similarly, an MIC of 2 mg/mL and MBC of 4 mg/mL against *Bacillus cereus*, *Salmonella Typhimurium*, and *Enterobacter cloacae* were reported. These effects could be attributed to this plant by-product’s phenolic compounds and other bioactive constituents. Archindia Velarde et al. [43] utilized guava leaf extract to determine the MIC against various bacterial strains, for example, *E. coli* (0.62 mg/mL), *Salmonella enterica* serotype ATCC 13076 (1.25 mg/mL), *Klebsiella* sp. (0.62 mg/mL), *Pseudomonas* sp., (0.62 mg/mL), *L. monocytogenes* (0.62 mg/mL), and *Staphylococcus* (0.62 mg/mL), which demonstrated a low-dose broad-spectrum inhibition. Pomegranate peel is renowned for its broad spectrum against bacterial pathogens, with an MIC against methicillin-resistant *S. aureus* (MRSA) reported to be 15.63 mg/mL and 31.25 mg/mL against *L. monocytogenes* [44].

It has been proposed that the antimicrobial activity of phenolic compounds against foodborne pathogens can be attributed to a possible cytoplasmic membrane-centric mechanism, specifically, hyper-acidification at the plasma membrane interface, derived from the dissociation of phenolic acids. This process can alter cell membrane potential, increasing its permeability, while the variable sensitivity to phenolic acids among pathogenic microorganisms could be explained by this mechanism [45]. It is important to note that Gram-positive bacteria, lacking an outer membrane, allow for an easier diffusion of phenolic acids through the cell wall, as compared to Gram-negative bacteria. In contrast, the outer membrane of Gram-negative bacteria acts as a barrier against hyper-acidification, which could explain the different level of resistance observed in Gram-negative bacteria. Phenolics and other compounds exhibiting antioxidant activity may also exert antimicrobial activity, although the bacterial species will always condition the latter. Possible structural properties that influence these bioactivities are the number and position of hydroxyl groups present in phenolics, specifically, their position in the aromatic ring and the length of the saturated side chain. These hydroxyl groups can interact with the bacterial cell membrane, causing the loss of cellular components and affecting the active site of enzymes, resulting in damage to some of their metabolic processes [46].

The antimicrobial activity of bioactive compounds in agro-industrial by-products represents a promising field for addressing significant food security and public health challenges. The inherent ability of these compounds to inhibit the growth and development of pathogenic microorganisms offers valuable opportunities in food preservation and the prevention of foodborne illness. The diversity of their bioactive compounds, such as total phenolics, flavonoids, and other phytochemicals, gives these by-products antimicrobial properties that can effectively fight bacteria and fungi. Not only does this approach help reduce food waste, it also addresses environmental concerns and presents innovative alternatives to improve food safety and quality. Continued research in this area could lead to identifying novel compounds with specific antimicrobial properties, thereby contributing to developing more effective strategies for food preservation and mitigating health risks. Ultimately, the exploration and application of the antimicrobial activity of bioactive compounds in agro-industrial by-products offer a path towards more sustainable and safe practices in food production. Table 2 summarizes the MIC and MBC of various bioactive compounds from by-products of plant origin [42,47,48,49,50,51].

## 4. Antioxidant Activity of Agro-Industrial By-Products of Plant Origin

The antioxidant activity of agro-industrial by-products of plant origin is closely linked to their bioactive compounds, according to their ability to neutralize free radicals and mitigate oxidative stress in the human body. Such antioxidant properties can help prevent various chronic diseases, including some types of cancer, diabetes, and cardiovascular disease [52]. Valorizing these by-products as sources of antioxidants therefore has positive implications for human health, while also promoting environmental sustainability by reducing the agri-food industry’s waste and the need for new raw materials [53]. This approach contributes to the circular economy by making the most of available resources and improving efficiency when using natural resources.

The bioactive compounds in agro-industrial by-products also play a crucial role in developing functional foods enriched with antioxidants. Not only do these foods offer additional nutritional benefits, they can also contribute to preventing diseases related to oxidative stress, providing healthier and more natural options to consumers. In addition, by integrating natural antioxidants into the formulation of food products, the need for synthetic additives is reduced, thus meeting the growing demand for more natural and sustainable food products [54].

Much research has been performed to explore the antioxidant activity of agro-industrial by-products, revealing their potential to be used as sources of antioxidant compounds. Such is the case of inferior peaches and peach kernel almonds that had significant antioxidant capacity, with IC_50_ values of 2.66 μg/mL and 7.88 μg/mL, respectively. Significant levels of phenolic compounds were also detected, with a total phenolic content of 253.4 mg GAE/100 g for the peaches and 29.3 mg GAE/100 g for the almonds. These findings highlight the relevance of these by-products as valuable sources of natural antioxidants with potential applications in the food industry and for health promotion [55].

Corn cobs subjected to multi-enzyme hydrolysis had a markedly superior antioxidant capacity as compared to that of non-hydrolyzed samples, according to 2,2-diphenyl-1-picrylhydrazyl (DPPH) and 2,2′-azino-bis(3-ethylbenzothiazoline-6-sulfonate) (ABTS) assays. Specifically, the hydrolyzed corn cob showed a concentration of 5.36 mg rutin equivalent (RE)/mL in the DPPH assay and 10.7 mg Trolox equivalent (TE)/mL in the ABTS assay. In contrast, the non-hydrolyzed corn cob had significantly lower values of 1.28 mg/mL and 1.64 mg/mL in the aforementioned assays. These findings highlight the enhanced antioxidant potential of corn cobs after a multi-enzyme hydrolysis, suggesting their usefulness as a natural source of antioxidants [56].

Evaluating the antioxidant activity of various agro-industrial by-products using the DPPH method has revealed the presence of bioactive compounds with a well-known antioxidant capacity. For example, detailed studies have shown by-products of grape pomace have a concentration of 87.8 μM TE/g and those of spent coffee have up to 89.8 μM TE/g. In contrast, tomato pomace has a concentration of 21.5 ± 0.0 μM TE/g, and red corn cob has a concentration of 48.9 μM TE/g. These results highlight the potential of said agro-industrial by-products as valuable sources of natural antioxidants [18].

By-products of ‘San Andreas’ and ‘Festival’ strawberry cultivars from Argentina have antioxidant activity values of 16.3 and 15.1 mmol TE/kg, respectively [3]. This shows that these by-products retain a potential source of antioxidants. ‘Cavendish’ banana peels (*Musa acuminata*) have an antioxidant activity of 1.2 mg ascorbic acid equivalent (AAE)/g, according to the DPPH method. In addition, values of 0.81 mg AAE/g were observed in a ferric reducing antioxidant power (FRAP) assay and 1.31 mg AAE/g in an ABTS assay, indicating significant antioxidant activity [57]. These findings highlight the potential of banana peels as natural sources of antioxidants, suggesting some potential applications.

An investigation was carried out on the antioxidant activity of flours obtained from tropical fruit residues. The results showed that acerola flour had the highest antioxidant activity, with an ABTS value of 3294.34 mg AAE/100 g, FRAP value of 8604.58 μmol ferrous sulfate equivalent (FSE)/100 g, and DPPH value of 2226.24 μmol TE/100 g. It was followed by umbu flour, with an ABTS value of 3083.95 mg AAE/100 g, FRAP value of 12,639.62 μmol FSE/100 g, and DPPH value of 2257.61 μmol TE/100 g. Genipap flour showed a moderate level of antioxidant activity, with an ABTS value of 583.52 mg AAE/100 g, FRAP value of 2126.52 μmol FSE/100 g, and DPPH value of 2222.08 μmol TE/100 g. Finally, guava flour exhibited the lowest antioxidant activity among those evaluated, with an ABTS value of 103.38 mg AAE/100 g d.w., FRAP value of 1144.05 μmol FSE/100 g, and DPPH value of 706.55.24 μmol TE/100 g (all units given in d.w.) [58]. These results highlight the variability in the antioxidant activity of tropical fruit flours. In a study by Rodrigues [59], the antioxidant activity of two by-products, cassava peel flour and malt bagasse, was investigated. The results showed that cassava peel flour exhibited an ABTS value of 26.31 μM TE/g, FRAP value of 0.02 μM FSE/g, and DPPH value of 24.13 μM TE/g. On the other hand, malt bagasse showed an ABT value of 35.73 μM TE/g, FRAP value of 0.32 μM FSE/g, and DPPH value of 4.32 μM TE/g. These findings indicate that both by-products have significant potential, due to the presence of bioactive compounds with antioxidant activity.

Two independent investigations revealed the remarkable antioxidant activity of seeds from different tropical fruits. On the one hand, açaí seed extract demonstrated antioxidant activity by the DPPH (622.81 ± 67.56 μmol/g) and ABTS (763.09 ± 17.27 μmol TEAC/g) free radicals [60]. On the other hand, a cupuassu seed (*Theobroma grandiflorum* Schum.) by-product exhibited significant antioxidant activity, as measured with ABTS (151.0 ± 5.5 mg TEAC/100 g crude extract) and DPPH (85.4 ± 1.7 mM TE/L) assays [61]. These findings highlight the potential of natural antioxidants present in tropical fruit seeds.

A study on Italian grape (*Vitis vinifera*) leaves and their antioxidant activity showed that ‘Gaglioppo’ leaf extract exhibited the highest radical scavenging capacity, with IC_50_ values of 7.19 μg/mL and 19.12 μg/mL in DPPH and ABTS assays, respectively. On the other hand, ‘Magliocco Dolce’ proved to be more effective against the DPPH radical, with an IC_50_ value of 12.47 μg/mL. ‘Magliocco Canino’ leaf extract had moderate radical scavenging activity, with IC_50_ values of 35.30 μg/mL and 31.02 μg/mL in DPPH and ABTS assays, respectively. The extract of ‘Arvino’ and ‘Greco Nero’ leaves showed less potent antioxidant activity, with IC_50_ values of 32.99 μg/mL and 86.33 μg/mL in DPPH and ABTS assays for ‘Arvino’ and 77.88 μg/mL and 78.85 μg/mL in DPPH and ABTS assays for ‘Greco Nero’ [62]. This research highlights the diversity of antioxidant values of tropical fruit seeds and Italian grape leaves. These results suggest that these by-products may exert good antioxidant properties. Table 3 summarizes the main compounds present in various agro-industrial by-products, as well as their antioxidant activity [63,64,65,66,67,68,69,70,71,72].

## 5. Therapeutic Uses of Bioactive Compounds Present in Agro-Industrial By-Products of Plant Origin

Research has shown that plant by-products generated from food processing, such as pomace, peels, and seeds, have a wide range of bioactive compounds in different concentrations [63]. Consumers are interested in products that have natural sources and provide health benefits; thus, bioactive compounds in by-products have been studied in vitro and in vivo in order to verify their potential therapeutic effects. The data have sparked growing interest; however, despite promising research, the number of clinical trials in humans is minimal. This section examines the positive effect of bioactive compounds, regarding their effects against some major non-communicable diseases. Additionally, potential mechanisms of action are provided, according to their use in animal studies. As with the antioxidant activity of by-products, their health-promoting effects and mechanisms of action are closely associated with the specific compounds present in them. For example, some compounds are able to produce an antioxidant environment that benefits the whole cell or tissue, while others may exert precise actions on certain key enzymes. This makes it necessary to consider both their bioactive profile and their potential mechanism of action in order to promote the use of by-products as sources of health-promoting compounds.

### 5.1. Dyslipidemia

Dyslipidemia is a metabolic disease defined by abnormal concentrations of total cholesterol, LDL, HDL, and/or triglycerides. Studies have shown that compounds found in agro-industrial by-products, such as gallic acid, may exert lipid-lowering properties. A meta-analysis indicated that supplementation with lutein could increase HDL levels in older adults (age ≥ 60); however, there are some limitations due to the low number of studies. The dose–response of lutein in its the lipid profile could not be evaluated, and the various health statuses of participants in the included trials may have affected the results, making it necessary to conduct more clinical trials to confirm these findings [73]. Animal studies have shown the effect of flavonoids in treating dyslipidemia; for example, Ahmad et al. [74] analyzed the effect of a flavonoid-rich beverage (500 mg/500 mL) (epigallocatechin gallate and catechins) in rats with dyslipidemia, showing a decrease in total cholesterol and LDL, suggesting its therapeutic potential.

Chao et al. [75] administered 205 mg/kg of gallic acid for 17 weeks to obese mice fed a high-fat diet. The authors reported that treatment with gallic acid prevented the downregulation of genes involved in lipid metabolism, such as sterol regulatory element binding protein (SREBP)-2, and positively regulated the expression of hydroxymethylglutaryl-CoA synthase (HMGCS). Another study reported the effects of gallic acid (100 mg/kg body weight) on markers of hepatic steatosis, lipogenesis, serum cholesterol, and triglyceride levels in obese mice for 8 weeks and determined that it decreased the mRNA expression of lipogenesis-associated genes (SREBP-1c and fatty acid synthase, FAS), as well as genes involved in the β-oxidation of fatty acids. According to these findings, gallic acid in agro-industrial by-products could have therapeutic potential against dyslipidemia.

Chlorogenic acid is another phenolic acid present in different by-products. Salamat et al. [76] conducted a randomized clinical trial in which participants consumed chlorogenic-acid-rich coffee extract supplements (800 mg/day) for 8 weeks, which led to a significant reduction in LDL. The authors theorized that the compounds present in the supplements influence LDL oxidation by reducing homocysteine, since homocysteine increases oxidation by promoting the formation of reactive oxygen species.

Luo et al. [77] reported the effects of ferulic acid in a murine model of mice fed a high-fat diet. The treatment reduced hypercholesterolemia, potentially due to the increased fecal excretion of bile acids. They also carried out studies to study its mechanism of action and showed that ferulic acid activated the expression of cytochrome 7A1 (CYP7A1, the rate-limiting enzyme in the biosynthesis of hepatic bile acids) through non-FXR (farnesoid X receptor) signaling, which prevents hypercholesterolemia, but may increase plasma bile acids. Wang et al. [78] studied the effects of sinapic acid (0.03%) on lipid metabolism in hamsters fed a high-fat diet for 12 weeks. A reduction in serum levels of total cholesterol, triglycerides, and LDL were observed, in addition to an increase in HDL. Their results suggested that treatment with sinapic acid regulated intestinal mRNA levels of Niemann-Pick C1-Like 1 protein (NPC1L1) and SREBP2, which reduced intestinal cholesterol absorption and attenuated serum and fecal cholesterol levels. These studies suggest that phenolic acids may significantly lower cholesterol levels, inhibit lipoprotein oxidation, and improve overall cardiovascular health. These effects are attributed to phenolic acids’ antioxidant and anti-inflammatory properties, which act synergistically to counteract the pathological processes associated with dyslipidemia. The relationship between phenolic acids and dyslipidemia has promising prospects in promoting cardiovascular health; however, more research and clinical trials are needed to validate and translate these findings into practical recommendations for managing dyslipidemia in clinical practice.

### 5.2. Diabetes

Diabetes is a metabolic disease that affects millions of people worldwide and represents a significant public health challenge. The search for innovative and sustainable approaches to address this disease has led to exploring previously untapped resources, such as compounds present in agro-industrial by-products [79]. Understanding how they interact with physiological processes related to diabetes may offer new strategies for developing functional foods and dietary supplements.

A meta-analysis suggested that a daily intake of at least four cups of tea, a beverage rich in flavonoids, may reduce the risk of type 2 diabetes [80]. Rahimifard et al. [81] reported the antidiabetic properties of gallic acid using rat embryonic fibroblast cells. The mechanism of gallic acid may be due to enhancing cellular glucose uptake by stimulating the phosphatidylinositol 3-kinase (PI3K)/p-Akt signaling pathway and translocating the GLUT1, GLUT2, and GLUT4 glucose transporters. Orsolic et al. [82] reported the antidiabetic effect of caffeic acid (50 mg/kg) in diabetic mice; caffeic acid is a potent antioxidant that effectively lowers serum glucose. Ferulic acid (50 mg/kg) was studied in order to determine its effects on diabetic rats for 8 weeks [83]; an improvement was observed in the rats’ glucose levels, and diabetes-induced adverse effects on their kidney tissue were reduced. Likewise, Mani et al. [84] observed the effects of *p*-coumaric acid (20 mg/kg) on oxidative stress and nephropathy in diabetic rats for 12 weeks. The authors noted that this compound acts on diabetes-induced lipid peroxidation and the activities of antioxidant enzymes (catalase, glutathione-S-transferase, and superoxide dismutase).

Altindag et al. [85] reported the effect of individual and combined treatments of sinapic acid (20 mg/kg/day) and ellagic acid (50 mg/kg/day) in diabetic rats for 28 days. They observed better results in the combined treatment, according to its antihyperglycemic effect exerted by inducing the synthesis and secretion of insulin from pancreatic β-cells. Raskovic et al. [86] observed that a resveratrol-based treatment improves glycemic control in diabetic rats, as well as LDL and triglyceride levels. Resveratrol can potentiate insulin action by reducing adiposity, inhibiting the expression of inflammatory genes and activating different kinases; it is also known for its antioxidant properties that can protect β-cells. Research on the interaction between phenolic compounds and diabetes offers an encouraging outlook and opens the door to novel, more personalized therapeutic approaches. However, additional studies, including long-term clinical trials, are needed to confirm and translate these findings into practical guidelines for the prevention and treatment of diabetes.

### 5.3. Cancer

Cancer remains one of the leading causes of morbidity and mortality globally, highlighting the need to identify innovative and practical approaches to address this complex disease. The effects of bioactive compounds like phenolics, flavonoids, carotenoids, and other phytochemicals on cellular processes related to carcinogenesis have been closely examined. In various foods, these compounds have demonstrated antioxidant, anti-inflammatory, and antiproliferative properties in preclinical studies, suggesting their potential impact on modulating cancer initiation, promotion, and progression. In doing so, these studies contribute to developing more informed and targeted strategies. In a prospective cohort study, a strong association was reported between reduced cancer mortality and consuming 500 mg/day of flavonoids in smokers and alcohol consumers. However, evidence from observational studies is still incomplete, studies on cancer are scarce, and their effects on patients are unknown. Although data from in vitro studies and animal models suggest that flavonoids can influence important cancer-related mechanisms, patient data are limited and inconclusive [87]; therefore, further research in this area is required [88].

Moccia et al. [89] reported that a carotenoid-enriched pumpkin extract has an antiproliferative effect on HG3 cells, according to a 40% delay in cell proliferation. The authors attribute this to the homeostatic mechanism involving a cellular metabolic alteration, which increases AMPK Thr172, the active form of AMPK, which is a critical step in the biochemistry of autophagy. Alazzouni et al. [90] observed that, in a mouse model of colon cancer, a ferulic acid treatment (50 mg/kg for 4 weeks) improved their histological structure; however, a further analysis is required to explain the mode of action of ferulic acid against colon cancer.

Studies have also been conducted with combined phenolic compounds. Sawata et al. [91] reported that resveratrol conjugated with two ferulic acids represses the 3D proliferation of HCT116 cells. They also observed the inhibition of the 3D proliferation of MCF7 human breast cancer cells. Jang et al. [92] reported microRNA expression profiles in SNU-16 cells treated with *p*-coumaric acid. Reflecting the complexity and promise of this ever-evolving area of research, scientific evidence suggests that certain compounds present in foods may play a crucial role in cancer prevention and treatment, highlighting the diversity of biological mechanisms by which these bioactive compounds may influence carcinogenesis.

### 5.4. Neuroprotective Effect

Given the complexity of the nervous system and the variety of challenges associated with neurological diseases, research on the neuroprotective effect of bioactive compounds has been explored for potential preventive and therapeutic strategies [93]. Few clinical trials have achieved positive results using natural compounds/extracts rich in flavonoids to treat neurodegenerative diseases. One study demonstrated that consuming flavonoid-rich citrus juice could dramatically improve blood flow to the brain in healthy young adults, suggesting it may generate benefits related to memory and learning by enhancing neuronal function and promoting neuronal protection and regeneration [94]. However, most studies have been designed to highlight general effects on neuroprotection, indicating the need for further research.

Baek and Kim [95] found that algae extracts (high in carotenoids) exhibited neuroprotective effects on neuronal cells under induced oxidative stress; for example, they improved cell viability and attenuated the formation of reactive oxygen species by positively regulating the expression of brain-derived neurotrophic factor (BDNF) and antioxidant enzymes by activating the TrkB/Akt pathway. Nakayama et al. [96] also observed that they reduce the production of reactive oxygen species. This is attributed to ferulic acid increasing BDNF by regulating the expression of microRNA-10b after hydrogen peroxide stimulation. Dou et al. [97] observed that when resveratrol was used on mice with cerebral artery occlusion, it promoted Th1/Th2 balance, decreased the expression of small intestinal pro-inflammatory cytokines through the modulation of Th17/Tregs and Th1/Th2 polarity changes mediated by the gut microbiota in small-intestine lamina propria and protected against the disruption of the blood–brain barrier.

The antioxidant and neuroprotective effects of caffeic acid regarding oxidative damage and transcriptional regulation were evaluated in rat cortical slices. A volume of 100 μM of caffeic acid prevented the loss of reducing capacity, cell damage, and oxidative damage; this is attributed to the binding activity of Nrf2/ARE that participates in the protective mechanisms evoked by caffeic acid in mammalian cortical tissue [98]. Verma et al. [99] reported the effect of sinapic acid (20 mg/kg) on cognitive impairment in rats by acting on the cortex and hippocampus and showing neuroprotective effects against oxidative stress, neuroinflammation, and cholinergic dysfunction. This evidence suggests that plant by-products may be a potential source of bioactive compounds with numerous health benefits. Table 4 summarizes the health effects of various compounds found in by-products of plant origin [100,101,102,103,104,105,106,107,108,109,110,111,112,113,114,115].

## 6. Trends in Food and Sustainability

**Figure 1:**
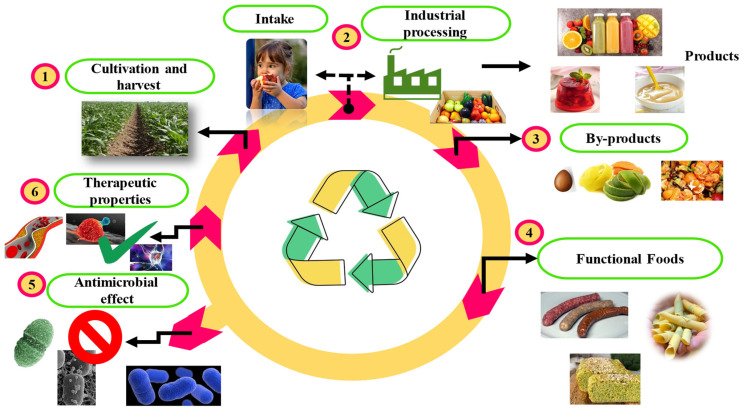
Proposed model for using plant by-products. Plants are cultivated and harvested (1) and either consumed fresh or industrially processed into various products (2); their industrial processing generates various by-products (3). These by-products can be used in functional foods (4) due to their antimicrobial (5) and therapeutic (6) potentials, thereby minimizing waste and contributing to a circular economy model.

There is interest in taking advantage of plant by-products, because they have been reported to be a valuable source of functional and nutritious ingredients. The search for new bioactive compounds is emerging as one of the most relevant challenges that will contribute to enhancing the alternatives of valorization and profitability to offer innovative products with potential effects on the health of consumers [122]. During this search, the application of the circular economy model has emerged in the development of innovative new products with bioactivities, as shown in Figure 1.

Most of the plant by-products of industrial processing are disposed of in landfills or inappropriate places, contributing to the proliferation of microorganisms which, on a large scale, becomes an environmental problem due to the production of greenhouse gases and toxic degradation products [123]. Several natural bioactive compounds can help prevent chronic diseases due to their mechanisms of action, such as their ability to interact with proteins, DNA, and other biological molecules [124].

Several investigations have been carried out in formulating products enriched with by-products from the food industry, standing out for their high content of compounds beneficial to health and their positive effects. Table 5 summarizes by-products that have been used to make different functional foods [125,126,127,128,129,130,131,132,133,134,135,136,137,138,139,140].

## 7. Future Prospects and Conclusions

Research on plant-based agro-industrial by-products reveals significant potential for developing sustainable solutions in various industries. Future studies must focus on optimizing bioactive compound extraction processes using green technologies, such as supercritical fluid extraction and natural solvents. In addition, integrating advanced bioprocessing techniques and waste biotransformation could improve compound recovery and functionality, thus increasing their commercial applicability and efficiency. Since higher percentages of vegetable by-product additions can alter the rheological and organoleptic properties of food products, the need to optimize the percentage of these additions should be emphasized.

In conclusion, plant-based agro-industrial by-products represent an opportunity to mitigate the environmental impact of waste and develop products with high added value, thanks to their antioxidant and antimicrobial properties. The bioactive compounds that they contain can potentially be used to prevent and treat various diseases, underscoring the importance of further research and promoting their use in therapeutic and nutraceutical formulations. The valorization of these by-products will contribute to environmental and economic sustainability and open up new avenues for innovation in multiple industrial sectors.

## Figures and Tables

**Table 1:** Content of bioactive compounds present in different agro-industrial by-products of plant origin.

Chemical Class	Bioactive Compounds	Concentration (µg/g d.w.)	By-Product	Ref.
Anthocyanin	Anthocyanin	147.4 ± 2.9	Cashew apple (peel and leftover pulp)	[36]
		9.0 ± 0.3	Guava (peel, leftover pulp, and seed)	
		22.9 ± 2.3	Mango (peel and leftover pulp)	
		37.0 ± 3.9	Passion fruit (seed)	
		101.0 ± 2.7	Pineapple (peel and leftover pulp)	
Carotenoids	β-carotene	1791.4 ± 179.2	Cashew apple (peel and leftover pulp)	[36]
		266.7 ± 26.7	Guava (peel, leftover pulp, and seed)	
		582.6 ± 58.3	Mango (peel and leftover pulp)	
		579.3 ± 58.0	Passion fruit (seed)	
		1561.0 ± 156.3	Pineapple (peel and leftovers pulp)	
Flavanols	Catechin	3640 ± 30	‘Hass’ avocado (seed)	[35]
		8130 ± 38	‘Fuerte’ avocado (seed)	
		132.27 ± 7.10	Bacaba (seed, peel, and pulp)	[32]
		53.6 ± 1.2	Grape (seed oil)	[30]
	Epicatechin	10,270 ± 80	‘Hass’ avocado (seed)	[35]
		11,060 ± 30	‘Fuerte’ avocado (seed)	
		73.4 ± 15.1	Achachairú (seed, peel, and pulp)	[32]
		1.67 ± 0.29	Aracá-boi (seed, peel, and pulp)	
		122.65 ± 4.89	Bacaba (seed, peel, and pulp)	
		1.29 ± 0.01	Avocado (peel)	[37]
		0.02 ± 0.0	Banana (peel)	
	Rutin	6.26 ± 0.04	Bacaba (seed, peel, and pulp)	[32]
		34.0 ± 7.0	Colombian acaí (pulp)	[33]
Flavanone glycosides	Naringenin	115.8 ± 1.1	Melon (peel)	[31]
		1.09± 0.31	Achachairú (seed, peel, and pulp)	[32]
		0.28 ± 0.0	Bacaba (seed, peel, and pulp)	
		5380.3 ± 182.3	Pummelo ‘Jiwei’ (flavedo)	[34]
		58.8 ± 27.9	Pummelo ‘Aolangbulangke’ (seed)	
Flavones	Apigenin-7-glycoside	293.4 ± 1.7	Melon (peel)	[31]
	Flavone	135.1 ± 3.2	Melons (peel)	[31]
	Luteolin	67.3 ± 1.8	Melons (peel)	[31]
		9.0 ± 3.0	Colombian acaí (pulp)	[33]
	Luteolin-7-glycoside	165.1 ± 1.5	Melon (peel)	[31]
Hydroxycinnamic acids	Caffeic acid	0.60 ± 0.0	Bacaba (seed, peel, and pulp)	[32]
		19.0 ± 0.80	Colombian acaí (pulp)	[33]
		0.43 ± 0.7	Pummelo ‘Jiwei’ (flavedo)	[34]
		3.65 ± 0.29	Pummelo ‘Aolangbulangke’ (seed)	
		7.9 ± 0.1	Grape (seed oil)	[30]
		6.9 ± 0.0	Passion fruit (seed oil)	
	Chlorogenic acid	82.5 ± 10.1	Melon (peel)	[31]
		0.79 ± 0.60	Bacaba (seed, peel, and pulp)	[32]
		33.57 ± 2.69	Pummelo ‘Jiwei’ (flavedo)	[34]
		7.57 ± 0.55	Pummelo ‘Aolangbulangke’ (seed)	
	*p*-coumaric acid	0.95 ± 0.10	Achachairú (seed, peel, and pulp)	[32]
		0.47 ± 0.02	Aracá-boi (seed, peel, and pulp)	
		0.56 ± 0.02	Bacaba (seed, peel, and pulp)	
		48.4 ± 0.4	Guava (seed oils)	[30]
		7.3 ± 0.4	Passion fruit (seed oil)	
		8.0 ± 0.1	Soursop (seed oil)	
	Ferulic acid	0.76 ± 0.01	Bacaba (seed, peel, and pulp)	[32]
		1.12 ± 0.11	Pummelo ‘Jiwei’ (flavedo)	[34]
		0.67 ± 0.04	Pummelo ‘Aolangbulangke’ (seed)	
	3-hydroxybenzoic acid	334.5 ± 3.7	Melon (peel)	[31]
	Isovanillic acid	237.0 ± 0.4	Melon (peel)	[31]
	Protocatechuic acid	34.6 ± 8.1	Melon (peel)	[31]
		2.78 ± 0.31	Bacaba (seed, peel, and pulp)	[32]
		17.0 ± 4.0	Colombian acaí (pulp)	[33]
	Syringic acid	48.0 ± 11.0	Colombian acaí (pulp)	[33]
Lignans	Pinoresinol	19.2 ± 0.7	Melon (peel)	[31]
Phenolic acids	Tyrosol	113.5 ± 0.3	Melon (peel)	[31]
Phenylethanoids	Hydroxytyrosol	91.1 ± 2.6	Melon (peel)	[31]
Tannins	Gallic acid	8.130 ± 1	Melon (peel)	[31]
		8.26 ± 0.17	Aracá-boi (seed, peel, and pulp)	[32]
		130.9 ± 9.6	Pummelo flavedo	[34]
		20.66 ± 2.58	Pummelo seed	
Tocopherols	α-tocopherol	11.8 ± 0.0	Grape (seed oil)	[30]
		45.8 ± 0.2	Guava (seed oil)	
		20.5 ± 0.3	Melon (seed oil)	
		7.3 ± 0.00	Pumpkin (seed oil)	
		22.1 ± 0.0	Soursop (seed oil)	
	ϒ-tocopherol	60.1 ± 0.1	Grape (seed oil)	[30]
		93.1 ± 0.2	Guava (seed oil)	
		249.6 ± 0.0	Melon (seed oil)	
		107.2 ± 0.2	Passion fruit (seed oil)	
		294.5 ± 0.3	Pumpkin (seed oil)	
		7.1 ± 0.0	Soursop (seed oil)	
		328.7 ± 0.3	Tomato (seed oil)	

**Table 2:** Minimum inhibitory (MIC) and bactericidal concentration (MBC) of agro-industrial by-product of plant origin.

By-Product	Bioactive Compound	Antimicrobial Activity against	Outcomes (mg/mL)	Ref.
Apple peels	Phenolic compounds	*Enterobacter faecium*	MIC = 15.6 MBC = 31.2	[47]
		*Escherichia coli*	MIC = 15.6 MBC = 31.2	
		*Listeria monocytogenes*	MIC = 62.5 MBC = 125.0	
		*Pseudomonas aeruginosa*	MIC = 15.6 MBC = 31.2	
		*Salmonella* *typhimurium*	MIC = 31.2 MBC = 62.5	
		*Staphylococcus aureus*	MIC = 3.9 MBC = 7.8	
Artichoke (*Cynara scolymus* L.) floral stems	Luteolin, apigenin derivaties,1-*O*-, 3-*O*, 4-*O*, and 5-*O*-caffeoylquimic acids, and procyanidin dimer	*Candida albicans*	MIC = 1.0 MBC = 1.0–2.0	[48]
		*Enterococcus faecium*	MIC = 1.0–1.5 MBC = 1.5–2.0	
		*Escherichia coli*	MIC = 1.0–1.5 MBC = 1.0–1.5	
		*S. typhimurium*	MIC = 1.0–1.5 MBC = 1.5–2.0	
		*S. aureus*	MIC = 1.0–1.5 MBC = 1.5–2.0	
Jaboticaba (*Myrciaria jaboticaba Vell. Berg*) peels	Bis-HHDP-glucose, galloyl-bis-HHDP-glucose, pentagalloyl glucose, trisgalloyl-HHPD-glucose, and bis-HHDP-glucose	*Enterococcus faecalis*	MIC = 10.0 MBC = > 20.0	[49]
		*E. coli*	MIC = 20.0 MBC = > 20.0	
		*Klebsiella pneumoniae*	MIC = 20.0 MBC = > 20.0	
		*Listeria monocytogenes*	MIC = 10.0 MBC = > 20.0	
		*Pseudomonas aeruginosa*	MIC = 20.0 MBC = > 20.0	
		MRSA	MIC = 10.0 MBC = > 20.0	
Kiwi (*Actinidia deliciosa* cv. ‘Hayward’)	Epicatechin, B-type (epi)catechin, and quercetin	*Bacillus cereus*	MIC = 2.0 MBC = 4.0	[42]
peels		*Enterobacter cloacae*	MIC = 2.0 MBC = 4.0	
		*E. coli*	MIC = 1.0 MBC = 2.0	
		*L. monocytogenes*	MIC = 2.0 MBC = 4.0	
		*S. typhimurium*	MIC = 2.0 MBC = 4.0	
		*S. aureus*	MIC = 1.0 MBC = 2.0	
Olive (*Olea europaea*) leaves and branches	Luteolin and tyrosol	*E. coli*	MIC = 40.0 MBC = 45.0	[50]
		*Listeria innocua*	MIC = 20.0 MBC = 25.0	
		*Salmonella* sp.	MIC = 35.0 MBC = 40.0	
		*S. aureus*	MIC = 20.0 MBC = 25.0	
Pomegranate (*Punica granatum* L.)	Phloretin, quercetin, indolamine, coutaric acid, isohydroxymatairesinol, and punicatannin C	*Staphylococcus epidermidis*	MIC = 0.1 MBC = 0.3	[51]
		*E. coli*	MIC = 0.3 MBC = 0.7	
		*Pseudomonas aeruginosa*	MIC = 0.1 MBC = 1.5	
		*E. faecalis*	MIC = 0.2 MBC = 0.7	

**Table 3:** Antioxidant activity of bioactive compounds present in agro-industrial by-products.

By-Products	Chemical Class	Method	Antioxidant Activity	Ref.
Avocado paste	Hydroxycinnamic acids	FRAP	3.52 ± 0.33 mg TE/g d.w.	[63]
		DPPH	1.57 ± 0.14 mg TE/g d.w.	
		ABTS	5.89 ± 0.34 mg TE/g d.w.	
Grape seed flour	Phenolic acids, flavonoids, and procyanidins	FRAP	225.23 ± 1.89 µmol TE/g	[64]
		DPPH	65.66 ± 5.03%	
Mango seed	Hydroxycinnamic acids	ABTS	10,568 ± 73.05 mg TE/100 g d.w.	[65]
		DPPH	10,659 ± 419.69 mg TE/100 g d.w.	
Unripe papaya powder	Carotenoids and hydroxycinnamic acids	FRAP	411.58 ± 38.0 mmol FeSO_4_/100 g	[66]
		DPPH	37.87 ± 3.69 mg ascorbic acid/100 g	
Pineapple peel	Flavonoids and hydroxycinnamic acids	DPPH	93.12 ± 0.43%	[67]
		ABTS	3.19 ± 0.02 mg TE/g d.w. extract	
		β-carotene blanching	5.74 ± 0.10%	
Pineapple core		DPPH	93.22 ± 3.11%	
		ABTS	3.07 ± 0.01 mg TE/g dry extract	
		β-carotene blanching	5.62 ± 0.02%	
Pineapple pomace		DPPH	27.03 ± 1.18%	
		ABTS	3.04 ± 0.01 mg TE/g d.w.	
		β-carotene blanching	5.51 ± 0.03%	
Raspberry pomace	Anthocyanins, ellagitannins	FRAP	772.73 ± 10.50 µmol/L	[68]
		DPPH	361.27 ± 5.65 µmol TE/100 g	
Apple pomace	Flavonoids and hydroxycinnamic acids	DPPH	9.75 ± 1.15 g ascorbic acid/kg d.w.	[69]
		FRAP	10.87 ± 0.26 g ascorbic acid/kg d.w.	
Coffee pulp extracts	Hydroxycinnamic acids and anthocyanins	ABTS	27.0 ± 1.2 IC_50_ μg/mL	[70]
		DPPH	140.0 ± 9.2 IC_50_ μg/mL	
Citrus juice by-products	Hydroxycinnamic acids and flavones	DPPH	11,035 ± 549 µmol TE/g d.w.	[71]
		ORAC	91.570 ± 12.153 µmol TE/g d.w.	
Chokeberry pomace	Hydroxycinnamic acids and anthocyanin	DPPH	301.89 ± NR μM TE/100 g d.w.	[72]
		ABTS	779.58 ± NR μM TE/100 g d.w.	

ABTS: 2,2′-azino-bis(3-ethylbenzothiazoline-6-sulfonate); DPPH: 2,2-diphenyl-1-picrylhydrazyl; FRAP: ferric reducing antioxidant power; ORAC: oxygen radical absorbance capacity; TE: Trolox equivalents; NR: not reported; d.w.; dry weight.

**Table 4:** Therapeutic uses of bioactive compounds of agro-industrial by-products of plant origin.

Therapeutic Uses	Compound	Model/ Intervention	Main Results	Ref.
Dyslipidemia	Gallic acid	Swiss male mice fed high-fat diet, 100 mg/kg/body weight, 60 days	Improved glucose tolerance and metabolic parameters. Bioinformatic analyses showed that SIRT1 is the main target in the thermogenesis process, which was confirmed by higher mRNA expression of SIRT1 in brown adipose tissue	[100]
	Chlorogenic acid	Lepr ^db/db^ mice, ip 250 mg/kg, 2 weeks	Inhibited hepatic glucose 6-phosphatase expression and activity, attenuated hepatic steatosis, improved lipid profile, skeletal muscle glucose uptake, fasting glucose, glucose tolerance, insulin sensitivity, and dyslipidemia	[101]
	Caffeic acid	C57BL/6 mice fed high-fat diet, 0.02 and 0.08% *w*/*w*, 6 weeks	Reduced plasma and liver triglyceride and cholesterol concentrations, increased phosphorylation of AMPK, and decreased acetyl carboxylase, a downstream target of AMPK, which are related to hepatic β-oxidation of fatty acids	[108]
	Ferulic acid	Diabetic female Wistar rats, 40 mg/kg, 45 days	Significantly reduced elevated plasma lipid and blood glucose	[109]
	Carotenoids	Male C57BL/6J apoE knockout mice, 25, 50 and 100 mg/kg, 24 weeks	Decreased serum total cholesterol, triglyceride, and LDL concentrations	[116]
	Flavonoids	Male Sprague–Dawley rats, 10 mg/kg BW/day, orally, 90 days	Reduced lipid levels in serum and tissues. Inhibited hepatic activity of 3-hydroxy-3-methylglutaryl coenzyme A reductase	[117]
Diabetes	Gallic acid	Male C57BL/6J mice fed high-fat diet, 70 mg/kg, orally, one month	Decreased visceral fat, fasting blood glucose, and fasting insulin	[110]
	Chlorogenic acid	Male ^db/db^ mice, ip 250 mg/kg, 15, 30, 60, and 90 min	An acute decrease in fasting blood glucose 10 min after the compound was administered; the effect persisted for a further 30 min	[111]
	Caffeic acid	In vitro, 3.68 and 4.98 μg/mL	Inhibited α-amylase and α-glucosidase activities	[112]
	Ferulic acid	Male OLETF rats fed high-fat diet, 10 mg/kg/day, oral gavage, 45 weeks	Decreased blood glucose and markers of oxidative stress	[113]
	Flavonoids	Diabetic C57BL/6 male mice, 50 mg/kg/day, oral, 7 weeks	Decreased hyperglycemia and hepatic glucose production and increased glucose oxidation in muscle	[118]
Cancer	Gallic acid	HeLa cells and HUVEC, 10–400 μM, 24 h	Inhibited growth of HeLa cells by apoptosis and necrosis	[114]
	Chlorogenic acid	Various cell lines, 25 or 50 μM, 6 days. Male NOD/SCID mice with xenographed tumors, ip 25–200 mg/kg/day, 30 days. Male Wistar rats with gliomas, ip 75 mg/kg	Inhibited tumor growth and prevented the development of new tumors	[115]
	Caffeic acid	HeLa cells, 0.5, 1, 2.5, 5, or 10 mM, 24 h	Significantly reduced cell proliferation, decreased levels of uncleaved caspase-3 and Bcl-2, and induced cleaved caspase-3 and p53	[102]
	Ferulic acid	HeLa and Caski cells, 2.0 mM, 48 h	Inhibited cell invasion by reducing MMP-9 mRNA expression	[103]
	Carotenoids	Prostate cancer cells (PC-3, DU 145, and LNCaP), 20 μmol/L, 72 h	Reduced cell viability by inducing apoptosis	[119]
	Flavonoids	Various cell lines, 0–100 μM, 24 h	Suppression of cell growth, induction of apoptosis, cell cycle arrest, and inhibition of cell invasion	[120]
Neuroprotective	Chlorogenic acid	Inbred male Charles foster albino rats, 10 mg/kg, intranasal	Significantly reduced the area of cerebral infarction as well as the expression of TNF-α, iNOS, and caspase-3	[105]
	Caffeic acid	Male Sprague–Dawley rats, 10 μmol/kg, 15 min	Effective for lipid peroxidation, antioxidant enzyme activity, and neuronal protection	[106]
	Carotenoids	HT-22 cells, 100 μg/mL, 24 h	Neuroprotective effects on oxidative stress-induced neuronal cells. Improved cell viability and attenuated the formation of intracellular reactive oxygen species (ROS) and apoptotic bodies in hippocampal neuronal cells	[95]
	Flavonoids	Rats with middle cerebral artery occlusion and four-vessel occlusion, 40 and 80 mg/kg, 7 days	Reduction in ischemic injury and protection of hippocampal and cortical neurons	[121]
	Ferulic acid	Male Wistar rats, ip 50 mg/kg, 4 weeks	Re-establishment of antioxidant enzymes, prevented glutathione depletion, inhibited lipid peroxidation, and reduced inflammatory mediators, like cyclooxygenase-2 and iNOS, and pro-inflammatory cytokines	[107]

ip: intraperitoneal; OLETF: Otsuka Long-Evans Tokushima Fatty; HUVEC: primary human umbilical vein endothelial cell; SIRT1: sirtuin 1; AMPK: AMP-activated protein kinase; MMP: matrix metalloproteinase; TNF-α: tumor necrosis factor-α; iNOS: inducible nitric oxide synthase.

**Table 5:** Main by-products used in the production of functional foods.

Food Group	Product	Fruit	By-Product Used	Addition (%)	Effects	Ref.
Dairy products	Yogurt	Apple	Pomace	3.0	Increased total phenolics, dietary fiber, firmness, viscosity, and cohesion	[125]
		Passion fruit	Husk and seed	2.0	Increased fiber (soluble and insoluble), mineral content (K, Mg, and Mn), and viscosity; changes in color parameters	[126]
		Blueberry	Pomace	0.7	Significant increase in anthocyanins, total phenolic content, antioxidant activity, conjugated linoleic acid, and sensory acceptance	[133]
		Pineapple	Peel	1.0	Significantly reduced the fermentation time of milk co-fermented with probiotic organisms and increased fiber content	[134]
		Carrot	Fiber	1.0 and 2.0	Increased fiber content	[135]
	Cheese	Apple	Pomace	3.0	Texture and flavor enhancer increases daily fiber intake	[136]
	Powdered milk	Grape	Seed extract	0.1, 0.5, and 1.0	Significantly improved antioxidant activity (FRAP, DPPH, and ABTS)	[137]
Meat products	Chorizo	Plantain	Peel	6.9 and 7.2	Reducing pig fat incorporation results in excellent sensory characteristics due to technological parameters and sensory acceptance	[138]
	Sausage	Cacao	Pod shell	1.5 and 3.0	Starch substitution showed emulsion stability, increased fiber content	[139]
	Fish meat sausages	Onion	Peel	1.0 and 2.0	Improved sensory properties, extended shelf life	[140]
	Sausage	Tomato	Pomace	0.5, 1.0, and 1.5	Reduced nitrite levels, antimicrobial properties	[127]
Cereals	Wheat bread	Grape	Pomace	5.0 and 10.0	Increased fiber and phenolic compounds	[128]
	Spaghetti	Grape	Pomace	50.0	High fiber, antioxidant activity, and nutritional benefit with good consumer acceptability	[129]
	Breadstick	Red grape	Pomace	5.0 and 10.0	High fiber and antioxidant content inhibited microbial growth	[132]
	Dough	Fig	Shell	10, 13, and 16	Increased total phenolics, flavonoids, and antioxidant activity; pH control and preservation of sensory quality during storage	[130]
		Broccoli	Leaves	5.0	Extended service life, improved mineral content and its appearance without compromising cooking, texture, or sensory characteristics	[131]

## Data Availability

No new data were created or analyzed in this study. Data sharing is not applicable to this article.
